# Vitamin D3 ameliorates podocyte injury through the nephrin signalling pathway

**DOI:** 10.1111/jcmm.13180

**Published:** 2017-06-29

**Authors:** Ourania Trohatou, Effie‐Fotini Tsilibary, Aristidis Charonis, Christos Iatrou, Garyfalia Drossopoulou

**Affiliations:** ^1^ Institute of Biosciences and Applications NCSR ‘Demokritos’ Athens Greece; ^2^ Center for Clinical, Experimental Surgery and Translational Research Biomedical Research Foundation Academy of Athens (BRFAA) Athens Greece; ^3^ Center for Nephrology G. Papadakis General Hospital of Nikea‐Pireaus Athens Greece

**Keywords:** Diabetic nephropathy, glomerulus, nephrin, podocalyxin, Vitamin D_3_, Vitamin D_3_ Receptor, high glucose, paricalcitol, STZ

## Abstract

Renal podocytes form the main filtration barrier possessing unique phenotype maintained by proteins including podocalyxin and nephrin, which are modulated in pathological conditions. In diabetic nephropathy (DN), podocytes become structurally and functionally compromised. Nephrin, a structural backbone protein of the slit diaphragm, acts as regulator of podocyte intracellular signalling with renoprotective role. Vitamin D_3_ through its receptor, VDR, provides renal protection in DN but limited data exist about its effect on podocytes. In this study, we used isolated rat glomeruli to assess podocalyxin and nephrin expression after treatment with the 1,25‐dihydroxyvitamin D_3_ analogue paricalcitol in the presence of normal and diabetic glucose levels. The role of 1,25‐dihydroxyvitamin D_3_ (calcitriol) and its analogue, paricalcitol, on podocyte morphology and survival was also investigated in the streptozotocin (STZ)‐diabetic animal model. In our *ex vivo* model, glomeruli exhibited high glucose‐mediated down‐regulation of podocalyxin, and nephrin, while paricalcitol reversed the high glucose‐induced decrease of nephrin and podocalyxin expression. Paricalcitol treatment enhanced VDR expression and promoted VDR and RXR co‐localization in the nucleus. Our data also indicated that hyperglycaemia impaired survival of cultured glomeruli and suggested that the implemented nephrin down‐regulation was reversed by paricalcitol treatment, initiating Akt signal transduction which may be involved in glomerular survival. Our findings were further verified *in vivo*, as in the STZ‐diabetic animal model, calcitriol and paricalcitol treatment resulted in significant amelioration of hyperglycaemia and restoration of nephrin signalling, suggesting that calcitriol and paricalcitol may provide molecular bases for protection against loss of the permselective renal barrier in DN.

## Introduction

Active vitamin D_3_ is an important hormone implicated in prevention and treatment of hyperparathyroidism in dialysis patients. Increasing amount of data suggests a beneficial role of vitamin D_3_ in chronic kidney disease (CKD), associated with increased survival of patients 1, 2. One of the most common causes of end‐stage renal disease is DN 3. Vitamin D may provide renoprotection in DN in part *via* the renin‐angiotensin system 4, 5, 6. DN is characterized by pathological changes, such as thickening of the glomerular basement membrane and mesangial expansion and by the clinical hallmark of proteinuria 7. The field of research on DN has mainly focused on podocytes which are important for maintaining glomerular permselectivity 8.

Interestingly, podocytes lose their negative surface charge or slit diaphragm integrity, leading to disruption of the filtration barrier and subsequently to DN 9, 10. The slit diaphragm, a specialized cell junction between adjacent podocytes, is required for functional glomerular filtration, and its central component is nephrin. The nephrin‐associated protein complex includes several molecules, for example CD2‐associated protein (CD2AP), podocin and zonula occludens‐1 (ZO‐1) 11. It should be noted that the slit diaphragm proteins nephrin, CD2AP and podocin, in addition to their structural functions, are able to initiate PI3K/AKT‐dependent signal transduction in glomerular podocytes 12. It is well known that PI3K/p‐Akt signalling pathway protects glomerular podocytes and ameliorates proteinuria 13, 14, 15. However, there is no direct evidence to date to conclusively demonstrate that activation of the PI3K/AKT pathway by nephrin in the podocyte protects against apoptosis.

The anti‐proteinuric activity of vitamin D_3_ analogue has been confirmed in a number of clinical trials in diabetic patients with CKD 16. The renoprotective property of vitamin D_3_ has been confirmed through studies with various animal models of kidney disease 17. Vitamin D receptor (VDR), a member of the nuclear receptor superfamily, mediates the biological activities of vitamin D_3_ by forming a heterodimer with retinoid X receptor (RXR) and binding to vitamin D response elements (VDREs) in the regulatory region of target genes to regulate gene expression 18.

For example, we reported that vitamin D_3_ and its analogue, paricalcitol, maintained and induced the expression levels of specific components of human podocytes *in vitro*, restoring structural and functional integrity in a VDR‐dependent manner 19. Paricalcitol is a selective VDR activator (VDRA) with minimal hypercalcemic effects that reduces proteinuria in DN 16.

In this study, we explore the therapeutic role of paricalcitol *in vivo* and *ex vivo*, in isolated rat glomeruli exposed to diabetic conditions and provide insights into the molecular mechanisms activated *via* paricalcitol and the nephrin‐PI3K/pAkt signalling pathway in podocyte injury.

## Materials and methods

### Experimental animals

Wistar rats (180–220 g) were used for all the studies performed. Rats were housed in the animal facility of NCSR ‘Demokritos’ (EL25 BIO 019020022) and were maintained with free access to water and rat chow with a 12‐hrs light–dark cycle. Experimental protocols were approved by the Institutional Animal Care, and all animal experimentations were carried out in agreement with the ethical recommendations of the European Communities Council Directive of 22 September 2010 (2010/63/EU), animal welfare assurance number: protocol nbr: 6464. All procedures were performed under phenobarbital anaesthesia.

### Antibodies and reagents

Goat polyclonal anti‐nephrin N20, mouse monoclonal anti‐VDR (D‐6) and rabbit polyclonal anti‐RXR (C‐20) antibodies were purchased from Santa Cruz Biotechnology. Rabbit polyclonal anti‐PODXL antibody was purchased from Thermo Fisher Scientific (MA, USA). Rabbit monoclonal anti‐p85α/PI3 kinase antibody was purchased from Millipore (MA, USA). Rabbit polyclonal anti‐Akt, rabbit monoclonal anti‐phospho‐Ser473 Akt, rabbit polyclonal anti‐cleaved caspase‐3 and anti‐PARP antibodies were purchased from Cell Signaling. Mouse monoclonal anti‐tubulin antibody was purchased from Sigma‐Aldrich Ltd., (Gillingham, UK) Fluorescent secondary antibodies, donkey anti‐rabbit Alexa Fluor 594, donkey anti‐goat Alexa Fluor 488, donkey antimouse Alexa Fluor 594 and donkey antimouse Alexa Fluor 488 were purchased from Molecular Probes, Invitrogen Thermo Fisher Scientific, MA, USA. STZ was purchased from Sigma‐Aldrich.

### Isolation and culture of rat glomeruli

Glomeruli isolation was carried out as described by Sharma et al. 20. Briefly, a high mid‐line incision on the abdomen exposed the abdominal cavity and total bilateral nephrectomy was performed to anesthetized animals. The kidney capsules were removed, and glomeruli were isolated following consecutive passage of the mashed cortexes through screens of 80‐ and 200‐mesh size. Glomeruli were recovered from atop the 200‐mesh screen into Dulbecco's modified Eagle's medium (DMEM; Biochrom Gmb, Berlin, Germany) supplemented with 10% foetal calf serum (FCS; Biochrom Gmb),15 mM HEPES (Biochrom Gmb), 5 mg/ml insulin‐transferrin‐sodium selenite (ITS; Sigma‐Aldrich), 50 nM dexamethasone (Sigma‐Aldrich), 2 mM glutamine (Biochrom Gmb) and 5 mM or 25 mM D‐glucose (Sigma‐Aldrich) and incubated at 37°C in a 5% (v/v) CO_2_ humidified chamber for 4 or 8 days. The isolated rat glomeruli were treated with 150 nM or 300 nM paricalcitol (VDRA; Abbott, Hellas (BGP, Attika, Greece)) for 4 days in presence of normal (5 mM) or high (25 mM) glucose levels.

### Western blotting and immunoprecipitation

For Western blotting, following appropriate treatment where applicable, samples were lysed in modified RIPA lysis buffer [(150 mmol/l NaCl, 50 mmol/l Tris‐HCl (pH 7.4), 1 mmol/l 129 Na2EDTA, 1% (v/v) Triton X‐100, 0.25% (w/v) sodium deoxycholate and 0.1% (w/v) 130 sodium dodecyl sulphate (SDS), 1 × PhosSTOP (cocktail of phosphatase inhibitors, Roche, Mannheim, Germany) and 1 × Protease inhibitors cocktail (Roche)]. Protein concentration was determined by the Bradford colorimetric assay (Pierce). Immunoblot analysis of cell lysates was performed as previously described 19. To ensure equal amounts of protein loading, the blots were stripped (Re‐Blot Plus Mild Solution; Millipore) and re‐probed with anti‐β‐tubulin monoclonal antibody. Blots were developed using the ECL detection system (Thermo Fisher Scientific Inc.) and the ImageQuant^™^ LAS 4000 (GE Healthcare, UK). Quantification was performed using ImageJ software (IJ 1.46R).

Immunoprecipitation of nephrin and nephrin‐associated proteins from lysates was performed using 400 μg of total protein from each sample of isolated rat glomeruli. Briefly, 400 μg of total protein from each lysate was incubated overnight at 4°C with anti‐nephrin N20 antibody. Immune complexes were captured with protein G‐agarose (Merck KGaA) for 4 hrs at 4°C, followed by three washes in ice‐cold PBS. Immune complexes were extracted with 60 μl boiling 2×Laemmli sample buffer containing β‐mercaptoethanol. Fifty microlitres of each supernatant fraction was processed for Western blot analysis.

### Immunocytochemistry and fluorescence labelling of isolated glomeruli

Glomeruli cultured on glass coverslips were fixed with 4% (w/v) paraformaldehyde in PBS pH 7.4, for 15 min. at room temperature. After 10 min. quenching with 50 mmol/lt NH4Cl, cells were permeabilized with 0.25% (v/v) Triton X‐100 in PBS for 10 min., washed with PBS and blocked with 1% (w/v) BSA in PBS for 120 min. at room temperature. Glomeruli were then incubated with anti‐nephrin N‐20 antibody, antip85α/PI3 kinase antibody, anti‐phospho‐Ser473 Akt, anti‐Akt, anti‐VDR and anti‐RXR antibody overnight at 4°C and finally with the appropriate fluorescent secondary antibodies for 1 hr at room temperature (all antibodies in PBS containing 1% (w/v) BSA). Finally, coverslips were washed with PBS, incubated with DAPI (0.7 μg/ml) in PBS for 5 min. and washed again with PBS before mounting them with Dako Fluorescent Mounting Medium. Fluorescent specimens were examined with a confocal laser‐scanning microscope (TCS SP5 Confocal System, Leica Mannheim, Germany). Images were obtained and processed with Adobe Photoshop CS4 version 11.0, software.

### *In situ* cell death detection—TUNEL assay

Isolated rat grown on glass coverslips was fixed with 4% (w/v) paraformaldehyde in PBS pH 7.4, for 30 min. at room temperature, permeabilized for 30 min. at room temperature with 0.1% Triton X in 0.1% sodium citrate (pH6.0) and finally incubated with 50 μl TUNEL reaction mixture (TdT enzyme and fluorochrome labelling solution) for 1 hr at 37°C in the dark [*In Situ* Cell Death Detection Kit, TMR red (Roche Diagnostics)]. Finally, the isolated glomeruli were washed with PBS and incubated with DAPI in PBS for 5 min., before mounting with Dako Fluorescent Mounting Medium (Dako). Specimens were examined with a confocal laser‐scanning microscope (TCS SP5 Confocal System; Leica). Images were obtained and processed with Adobe Photoshop CS4 version 11.0, software.

### Animal treatment and design

To establish the diabetic model, STZ (Sigma‐Aldrich) dissolved in 0.1 mol/l citrate buffer (pH 4.5) at 65 mg/kg was single‐intraperitoneally injected (*n* = 15), while control rats received only the citrate buffer solution (*n* = 5). Blood glucose levels were derived from the caudal vein and measured 72 hrs after the injection using the Contour Link Meter (Bayer Diabetes, Canada). The model standard was confirmed after blood glucose measurements greater than 400 mg/dl. After 1 week of acclimatization, rats were randomly divided into four groups: control (C) (*n* = 5), STZ diabetic (DN) (*n* = 5), STZ rats treated with 400 ng/kg paricalcitol daily (DN‐P) (*n* = 5) and STZ rats treated with 100 ng/kg calcitriol daily (DN‐C) (*n* = 5). All animals were sacrificed at 5 weeks after STZ injection. At the end of the experiment and prior to sacrifice, bodyweights, blood samples and 24‐hrs urine samples were collected in metabolic cages. The kidneys were used for glomeruli isolation and for histological assessments.

### Histopathological analysis

Kidneys were fixed in 10% formalin and embedded in paraffin for histological investigation. Kidney sections were stained with sirius red reagent (Sigma‐Aldrich) for collagen determination and examined by light microscopy in a blinded manner. We analysed randomly selected non‐overlapping stained glomeruli from each group.

### Statistics

Experiments were performed at least three times, and results were expressed as means ± S.D. The Student's *t*‐test method was used to determine the statistical significance, and the *P*‐values are indicated in the figures, where (*) represents *P* < 0.05, (**) represents *P* < 0.01 and (***) represents *P* < 0.001. Statistical analysis for assessment of VDRA treatment in DN animal model was performed by one‐way anova with Greenhouse‐Geisser correction using Graph Prism version 7 (GraphPad software, CA, USA).

## Results

### VDRA enhanced the expression levels of podocyte markers and VDR in isolated glomeruli

**Figure 1:**
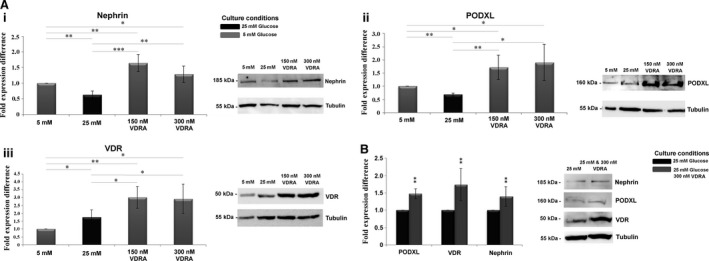
VDRA increased the expression levels of Nephrin, PODXL and VDR in isolated rat glomeruli. (**A**) Western blot analysis in isolated rat glomeruli cultured in 5 mM or 25 mM glucose in presence of 150 nM or 300 nM VDRA (paricalcitol) for (i) nephrin, (ii) PODXL and (iii) VDR. (**B**) Western blot analysis in isolated rat glomeruli cultured in 25 mM glucose or in 25 mM glucose in presence of VDRA for nephrin, PODXL and VDR. Western blot quantification was performed using ImageJ software, and the results were normalized to the tubulin positive control and then to isolated rat glomeruli cultured in 5 mM glucose. The values are presented as the mean ± S.D. of three independent experiments (*P < 0.05; **P < 0.01; ***P < 0.001 Student's *t*‐test).

Accumulating evidence suggests that VDRA, such as paricalcitol, can restore glucose‐mediated down‐regulation of nephrin and podocalyxin (PODXL) expression in T‐SV40‐Immortalized Human Glomerular Epithelial Cells 19. In an effort to determine whether VDRA may also affect functioning glomeruli, we analysed nephrin, PODXL and VDR expression levels in isolated rat glomeruli cultured in normal (5 mM) or diabetic (25 mM) glucose levels in the presence of 150 or 300 nM paricalcitol. Glomeruli cultured in the presence of 5 mM glucose displayed enhanced nephrin and PODXL expression levels when they were exposed to VDRA (Fig. 1A). Diabetic glucose levels induced a statistically significant decrease in nephrin and PODXL protein levels in isolated glomeruli (Fig. 1A). Interestingly, we observed that in glomeruli exposed to 25 mM glucose, VDRA increased the expression levels of nephrin and PODXL which were apparently restored to normal levels (Fig. 1B). In a similar pattern, VDRA enhanced VDR expression levels in either glucose condition (Fig. 1A and B).

### VDRA induced nuclear translocation of VDR and co‐localization with RXR

**Figure 2:**
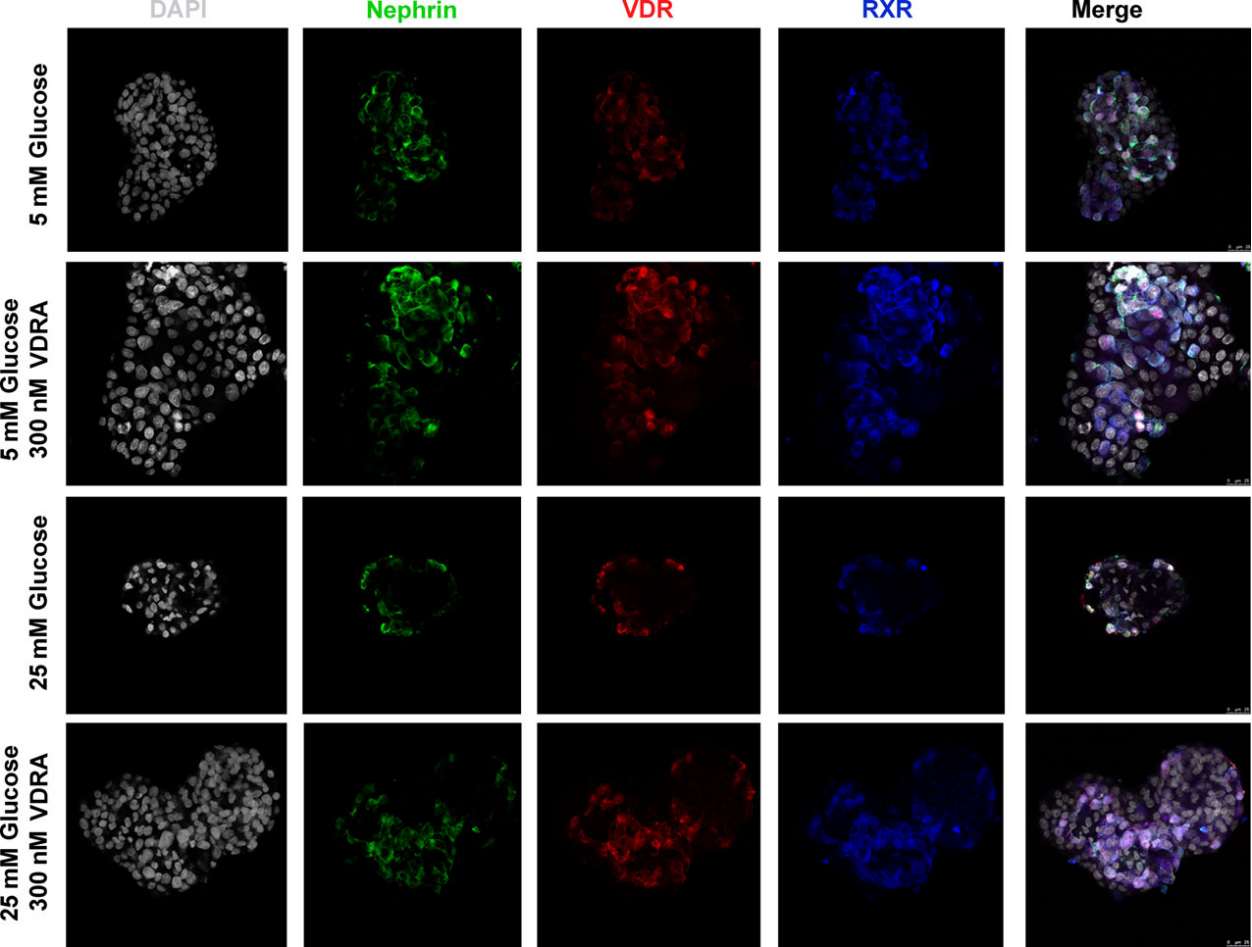
VDRA induced nuclear translocation of VDR and co‐localization with RXR. Representative confocal microscopy images of triple staining for Nephrin (green), VDR (red), RXR (blue) and DAPI (grey) in isolated rat glomeruli cultured in 5 mM glucose, 25 mM glucose and in presence of 300 nM VDRA. Original magnifications, 63×.

The subcellular localization of VDR and RXR in podocytes was analysed by immunofluorescence analysis in isolated rat glomeruli cultured in normal or hyperglycaemic conditions in presence or absence of 300 nM VDRA. In both normal and high glucose levels, these proteins partly co‐localized mainly in the nucleus (Fig. 2). Treatment with VDRA greatly enhanced VDR and RXR co‐localization in the nucleus (Fig. 2). Additionally, immunofluorescence staining of nephrin was performed, to verify that the VDR‐expressing cells were podocytes.

### High glucose impaired nephrin‐mediated PI3K‐pAkt signalling pathway

**Figure 3:**
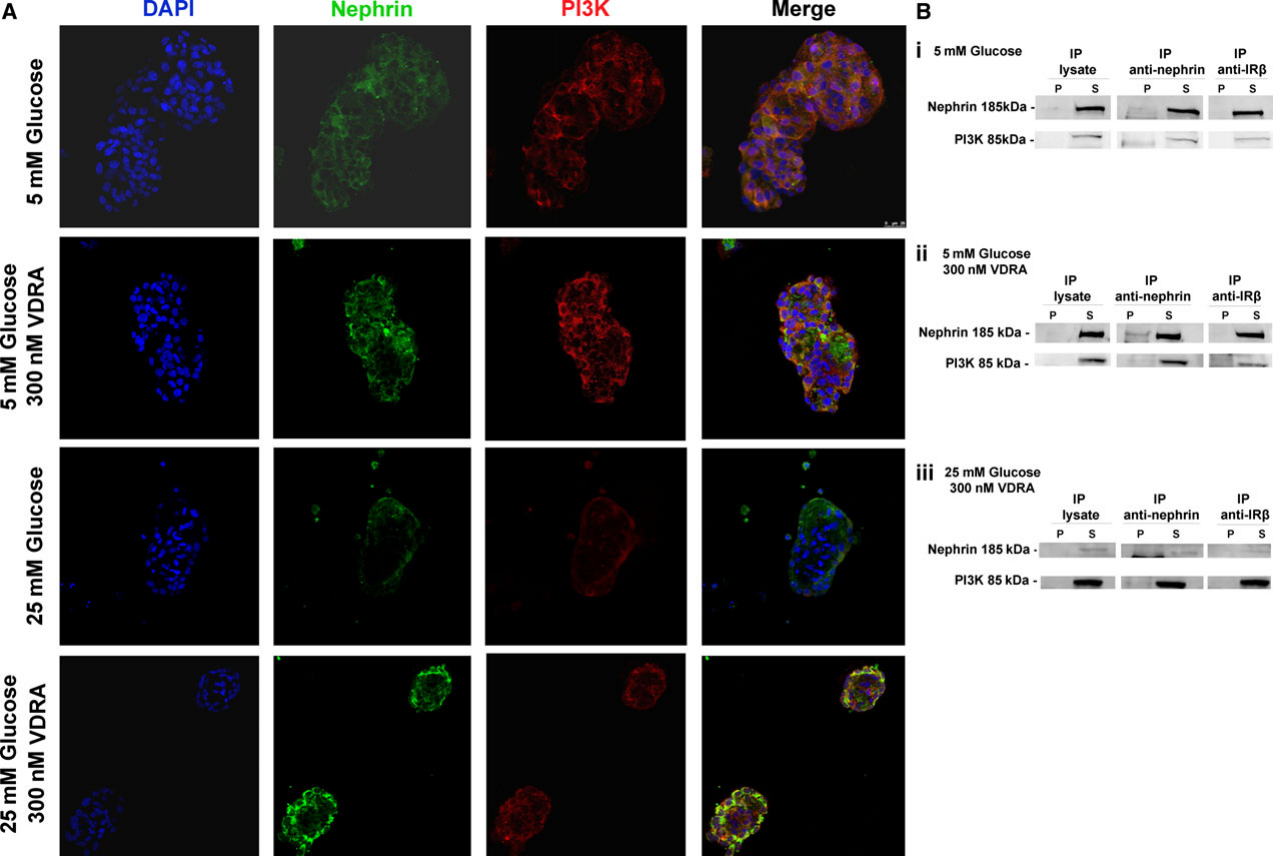
Nephrin and PI3K are co‐immunoprecipitated in isolated rat glomeruli lysate. (**A**) Representative confocal microscopy images of double staining for Nephrin (green), PI3K (red) and DAPI (blue) in isolated rat glomeruli cultured in 5 mM glucose, 25 mM glucose and in presence of VDRA. Original magnifications, 63×. (**B**) Immunoprecipitation (IP) with anti‐nephrin in isolated rat glomeruli cultured in (i) 5 mM glucose, (ii) 5 mM glucose in presence of VDRA and (iii) 25 mM glucose in presence of VDRA. No interactions were found with anti‐CBP, serving as negative control. P, precipitate; S, supernatant.

**Figure 4:**
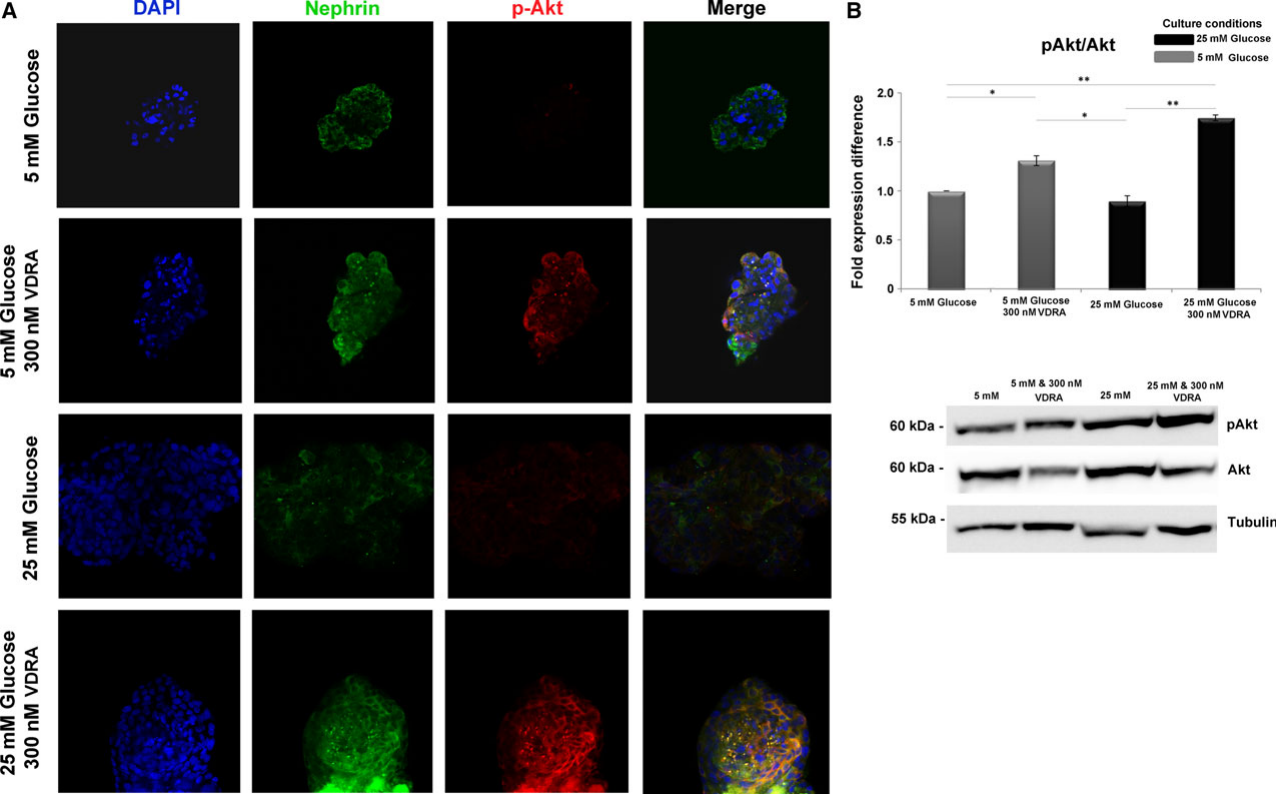
Paricalcitol enhanced Akt phosphorylation. (**A**)Representative confocal microscopy images of double staining for nephrin (green), pAkt (red) and DAPI (blue) in isolated rat glomeruli cultured in 5 mM glucose, 25 mM glucose and in presence of VDRA. Original magnifications, 63×. (**B**) Western blot analysis of pAkt/Akt in isolated rat glomeruli cultured in 5 mM glucose, 25 mM glucose and in presence of VDRA. Western blot quantification was performed using ImageJ software, and the results were normalized to the tubulin positive control and then to isolated rat glomeruli cultured in 5 mM glucose. The values are presented as the mean ± S.D. of three independent experiments (*P < 0.05; **P < 0.01 Student's *t*‐test).

Isolated glomeruli exhibited glucose‐dependent down‐regulation of nephrin expression. In view of the fact that nephrin signalling in podocytes is mediated *via* PI3K activation, we investigated the association of nephrin with the p85 regulatory subunit of PI3K in isolated glomeruli. Immunofluorescence co‐localization studies with nephrin and p85α‐PI3K in normo‐glycemic and hyperglycaemic glomeruli showed that nephrin is partially co‐localized with p85α‐PI3K after treatment with VDRA (Fig. 3A). These findings were further verified by co‐immunoprecipitation experiments with nephrin‐specific antibodies that showed that endogenous nephrin was associated with the p85α regulatory subunit of PI3K (Fig. 3B). We next examined whether this association may trigger PI3K‐dependent Akt signalling. Western blot analysis revealed that phosphorylation of Akt was significantly reduced in the presence of diabetic culture levels compared to glomeruli exposed to normal glucose conditions (Fig. 4A and B). Isolated glomeruli exposed to VDRA, exhibited a twofold increase of Ser473 Akt phosphorylation compared to non‐treated glomeruli (Fig. 4A and B), whereas basal Akt levels remained unaltered (Fig. S1). These results indicated that VDRA mediated enhancement of nephrin expression, may participate in PI3K activation and Akt phosphorylation in rat glomeruli *ex vivo*, thus inducing activation of the PI3K‐Akt signalling pathway.

### VDRA rescues glomeruli from glucose‐induced apoptosis

**Figure 5:**
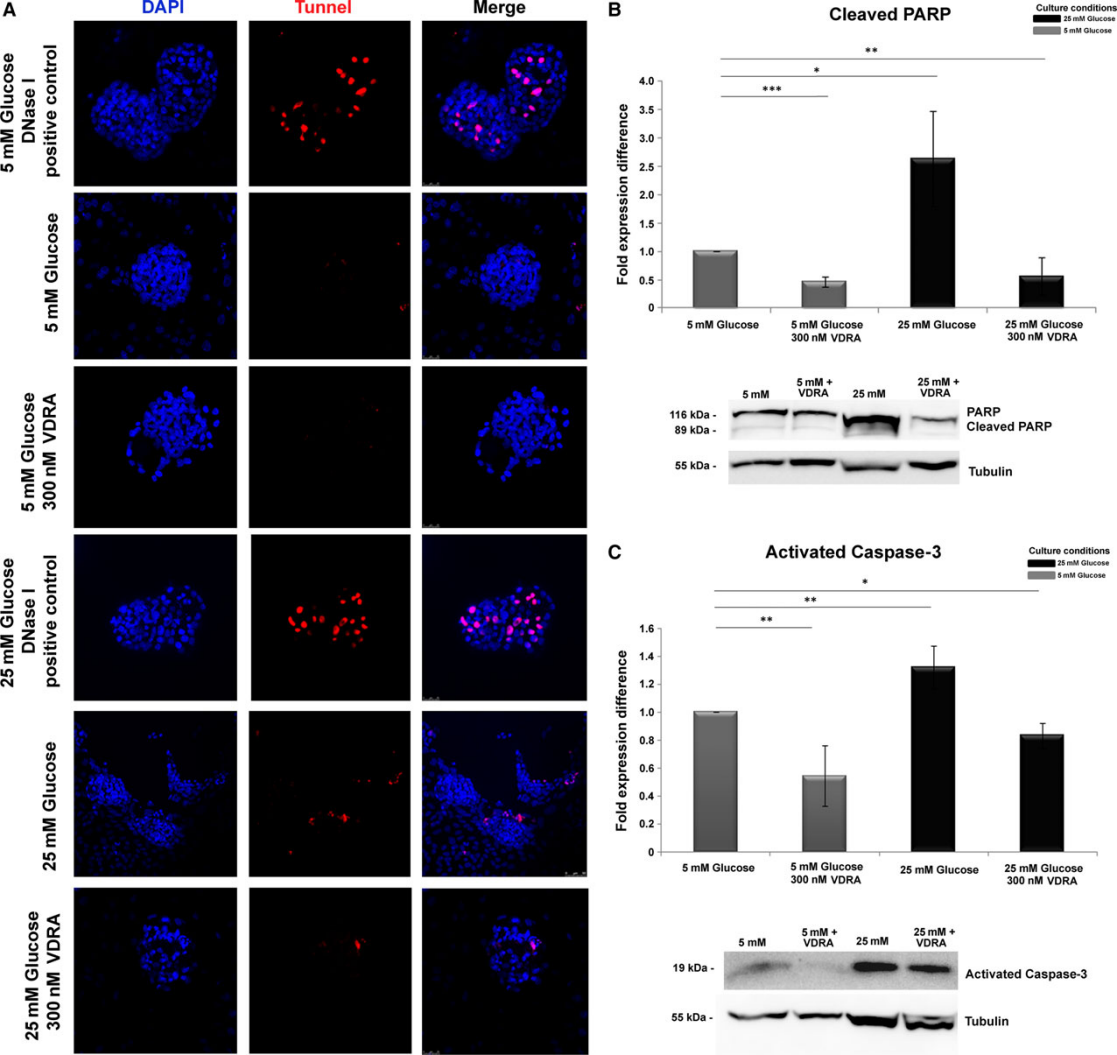
Determination of apoptosis in isolated rat glomeruli by TUNEL assay. (**A**) Representative fluorescent images of TUNEL‐positive cells in isolated rat glomeruli cultured in 5 mM glucose, 25 mM glucose and in presence of VDRA. Original magnifications, 63×. (**B**) Western blot analysis of cleaved PARP in isolated rat glomeruli cultured in 5 mM glucose, 25 mM glucose and in presence of VDRA. (**C**) Western blot analysis of activated caspase‐3 in isolated rat glomeruli cultured in 5 mM glucose, 25 mM glucose and in presence of VDRA. Western blot quantification was performed using ImageJ software, and the results were normalized to the tubulin positive control and then to isolated rat glomeruli cultured in 5 mM glucose. The values are presented as the mean ± S.D. of three independent experiments (*P < 0.05; **P < 0.01; ***P < 0,001 Student's *t*‐test).

In sequence, we investigated the effect of VDRA on cell survival. Caspase‐3 activity was monitored by caspase‐processing and cleavage of its downstream target, poly (ADP‐ribose) polymerase (PARP). Detection of caspase‐mediated cleavage of PARP has been established as a hallmark of apoptosis 21. Western blotting analysis demonstrated that upon exposure to 25 mM glucose levels, isolated glomeruli displayed increased levels of the large fragment (19 kD) of activated caspase‐3 and increased ratio of cleaved/uncleaved PARP, compared to glomeruli grown in the presence of 5 mM glucose levels (Fig. 5B and C). In addition, the degree of apoptosis was evaluated by detection of DNA strand breaks (*in situ* TUNEL and DAPI staining). Analysis of glomerular cells for DNA fragmentation (TUNEL‐positive cells) revealed that no TUNEL‐positive cells were observed in isolated glomeruli cultured in normal glucose levels with or without VDRA (Fig. 5A). However, increased glucose concentration resulted in significant increase in the number of TUNEL‐positive cells (Fig. 5A). This glucose‐induced susceptibility to apoptosis was inhibited by VDRA treatment (Fig. 5A). Taken together these findings clearly confirm the pivotal role of VDRA on cell survival.

### *In vivo* administration of VDRA in STZ animal model

To investigate the effect of VDRA on glomerular function *in vivo*, we performed studies in the streptozotocin‐induced (STZ) diabetic animal model. This is a widely used model for diabetes that has been employed for assessing the mechanisms of diabetes as well as screening potential therapies for the treatment of this condition, and evaluation of therapeutic options.

**Figure 6:**
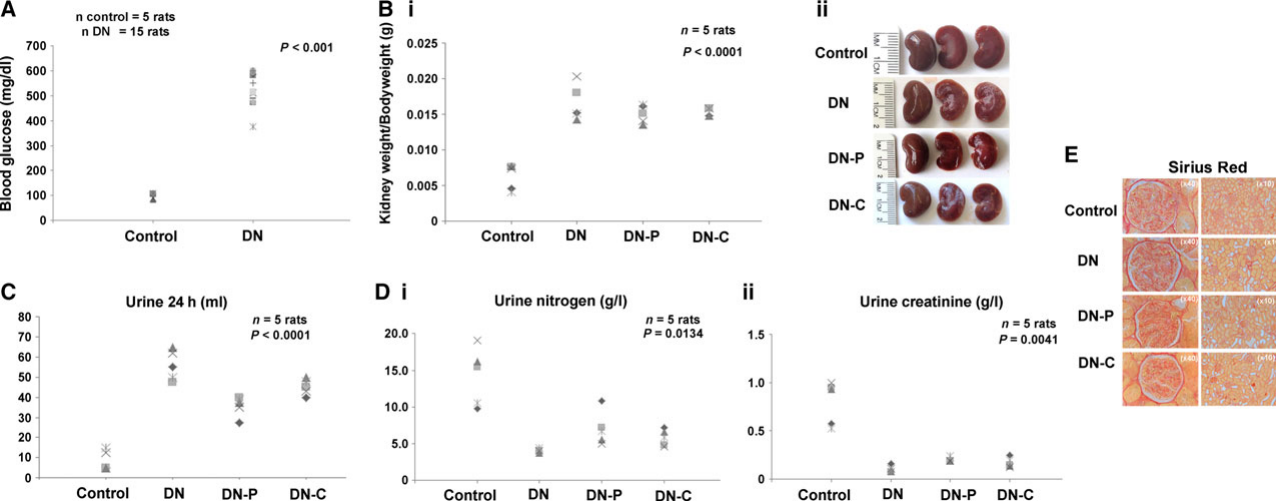
STZ model animals. Rats were randomly divided into four groups: control (C) (*n* = 5), STZ diabetic (DN) (*n* = 5), STZ rats treated with 400 ng/kg paricalcitol daily (DN‐P) (*n* = 5) and STZ rats treated with 100 ng/kg calcitriol daily (DN‐C) (*n* = 5). (**A**) Blood glucose levels (mg/ml) of control (C) and STZ‐diabetic animals (DN). (**B**) (i) Kidney weight/Bodyweight, (ii) Representative images of kidneys of control, STZ and diabetic animals after *in vivo* treatment with paricalcitol (DN‐P) or calcitriol (DN‐C). (**C**) Volume of urine 24 hrs (**D**) (i) urine nitrogen (g/l), (ii) urine creatinine (g/l). (**E**) Representative images of sirius red staining of C, DN, DN‐P and DN‐C animals. Original magnifications, 40× and 10×.

To assess the renal protective effect of VDRA, fasting blood glucose levels and bodyweights were measured starting from 3 days after STZ injection. As shown in Figure 6A, the rats in the DN group demonstrated elevated blood glucose levels, which were significantly higher than those of the non‐diabetic, control rats (*P* < 0.001). The kidney/bodyweight of the DN rats presented a significant increase compared to control rats (Fig. 6Bi, ii). During the study period, this increase was slightly abated in the DN‐P rats, but still remained elevated compared to non‐diabetic, control rats (Fig. 6Bi, ii). Interestingly, the 24‐hrs urine levels of DN‐P and DN‐C rats were significantly improved, in comparison with the DN group (*P* < 0.0001; Fig. 6C). Additionally, general parameters, namely urine creatinine and urine nitrogen, were also estimated. Specifically, evidence of VDRA‐induced podocyte protection was obtained from the urine nitrogen and urine creatinine levels that were significantly improved in comparison with the DN group (Fig. 6Di, ii). The above data suggest that VDRA treatment significantly reduced proteinuria and protected podocytes in the rats with experimentally induced diabetes.

### Effect of VDRA on histological changes

Using light microscopy, we evaluated the efficacy of VDRA in controlling DN in STZ rats. Glomeruli of STZ rats demonstrated increased nearly onefold accumulation of sirius red‐positive extracellular matrix proteins and the glomerular surface area became larger compared with that of normal rats. The above‐mentioned morphological kidney lesions were significantly attenuated after VDRA treatment (Fig. 6E).

### VDRA induces changes in the expression levels of nephrin and VDR in the glomeruli of rats with DN

**Figure 7:**
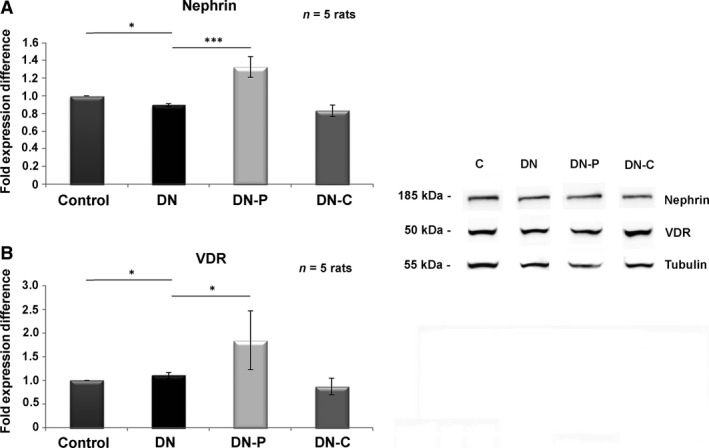
*In vivo* assessment of nephrin and VDR expression levels. Western blot analysis in isolated rat glomeruli after 5 weeks *in vivo* treatment with paricalcitol or calcitriol for (**A**) nephrin and (**B**) VDR. Western blot quantification was performed using ImageJ software, and the results were normalized to the positive control and then to isolated rat glomeruli from control animals, respectively. The values are presented as the mean ± S.D. of three independent experiments (*P < 0.05; ***P < 0.001 Student's *t*‐test).

Western blot analysis revealed a reduction in the expression of nephrin and VDR proteins in the glomeruli of rats with DN (Fig. 7). Following administration of VDRA, the protein levels of nephrin (Fig. 7A) and VDR (Fig. 7B) were markedly restored.

## Discussion

Active vitamin D is an important hormone implicated in prevention and treatment of hyperparathyroidism in dialysis patients. Increasing amount of data suggests a beneficial role of vitamin D in CKD, associated with increased survival of patients 1, 2. Vitamin D is important for ameliorating CKD and has been reported to reduce proteinuria in diabetic patients. Vitamin D regulates gene expression through its receptor, VDR, which interacts with VDREs located within regulatory regions of target genes. Mouse podocytes *in vitro* and *in vivo* and rat podocytes *in vivo* express VDR constitutively 22, 23, 24, 25. Our results provided evidence that VDR is also constitutively expressed *ex vivo*, in isolated rat glomeruli, and demonstrated for the first time that in our *ex vivo* model VDR expression and transport to the nucleus were affected by hyperglycaemia and were increased after VDRA treatment. In glomeruli cultured in high glucose levels, a modest but consistent increase of nuclear VDR was observed, compared to glomeruli cultured in normal glucose levels. This glucose‐mediated increase of VDR has also been reported in mouse podocytes *in vitro* 22. Glucose has been reported to interact with VDR resulting in impairment of its DNA binding and function within cells 26. Therefore, glucose‐mediated VDR increase in our *ex vivo* model could represent a compensatory mechanism against the hyperglycaemia‐induced inhibition of VDR binding on VDRE. This could also explain the fact that VDR expression levels in the STZ‐induced diabetic rats displaying DN were significantly decreased, suggesting that the compensatory mechanism is not applied in the *in vivo* situation.

It is known that VDR and RXR form heterodimers in the absence of VDRA regulating the basal transcriptional activity of target genes 27. In accordance with other findings we herein demonstrated that addition of VDRA stabilizes VDR‐RXR heterodimers and promote translocation of VDR to the nucleus. In accordance with our results, other reports have demonstrated that VDR is partly located in nuclei even in the absence of calcitriol and becomes accumulated to a greater extent in nuclei following addition of the ligand 19, 28. One important protein for sustaining normal podocyte structure and function is podocalyxin 29. Podocalyxin was permanently suppressed in immortalized human podocytes cultured in high glucose levels and this suppression was impeded following VDRA treatment 19, 30. We herein report for the first time that in isolated glomeruli VDRA managed to relatively restore the hyperglycaemia‐induced loss of podocalyxin, thus exerting a significant beneficial effect by maintaining and additionally restoring the differentiated podocytic phenotype. In our previous work, we have suggested that VDR is not necessarily associated with maintaining podocalyxin expression in normal conditions. However, VDR becomes apparently pivotal for re‐starting the expression of podocalyxin which has been previously suppressed by high glucose; this effect could be directly mediated by VDR or by a transcriptional complex of which VDR is a part 19. It has been also suggested that advanced glycation end products (AGEs) are partly involved in glucose‐dependent podocalyxin down‐regulation, since aminoguanidine (which prevents AGE formation), preserved podocalyxin expression 30. Vitamin D also moderates the deleterious impact of AGEs on endothelial cells by preventing the AGE‐induced elevation of NF‐kB‐p65‐DNA binding activity 31. Hence, an additional protective effect of VDRA in podocytes could be through moderation of AGE‐induced adverse effects or inhibition of AGE formation.

Nephrin is a cell surface receptor participating in cell–cell adhesion and signalling functions in glomerular podocytes 32. The present study focused on the role of VDRA on nephrin signal transduction in isolated rat glomeruli. We demonstrated that nephrin interacted/associated with the p85α regulatory subunit of PI3K *ex vivo* in isolated glomeruli. Similar interactions were also reported to regulate renal podocyte function *in vitro* 33. In our model PI3K association with nephrin resulted in activation of PI3K, as shown by activation of Akt phosphorylation. Phosphorylation of the intracellular domain of nephrin mediates interactions with several proteins, including Neph1, podocin, CD2AP, PI3K, that transmit nephrin signalling to the actin cytoskeleton and regulate several podocytic functions including survival 34, 35. The significance of nephrin signalling *in vivo* was previously well documented in glomerular podocytes. Specifically, CD2AP was reported to interact with both nephrin and PI3K *in vivo*, stimulating anti‐apoptotic Akt signalling in podocytes 12. Chronic hyperglycaemia disrupts signal transduction pathways involved in glomerular survival 36. Our previous work 37, as well as work by others 38 demonstrated a decrease of glomerular nephrin expression as an early event in hyperglycaemic conditions. We demonstrated herein that short‐term exposure of isolated glomeruli to high glucose affected nephrin expression and impaired phosphorylation and Akt activation.

Our data showed that nephrin expression was up‐regulated following VDRA treatment in glomeruli exposed to normal glucose levels. Similarly, immunofluorescence and Western blot studies revealed that glomeruli cultured in the presence of high glucose exhibited reduced total and cell surface nephrin expression. However, treatment with VDRA restored nephrin expression to levels similar to those observed in glomeruli cultured in normal glucose concentration. These findings were further verified by Western blot studies in glomeruli of STZ‐treated rats revealing and an *in vivo* reduction of nephrin expression which was rescued by VDRA treatment of the STZ‐treated rats. Additional studies have also indicated that vitamin D increased nephrin *in vivo* in murine and human podocytes 19, 39, 40, 41 and in rats with puromycin aminonucleoside‐induced nephrosis 42, 43, 44. Furthermore, paricalcitol increased nephrin expression *in vivo*, in adriamycin‐induced nephropathy 45 as well as in STZ‐treated rats 46. However, we herein demonstrate for the first time that hyperglycaemia‐induced nephrin induction is an early event in STZ‐induced DN, established within 5 weeks and VDRA treatment, can rescue nephrin expression at the initial stages of DN establishment.

**Figure 8:**
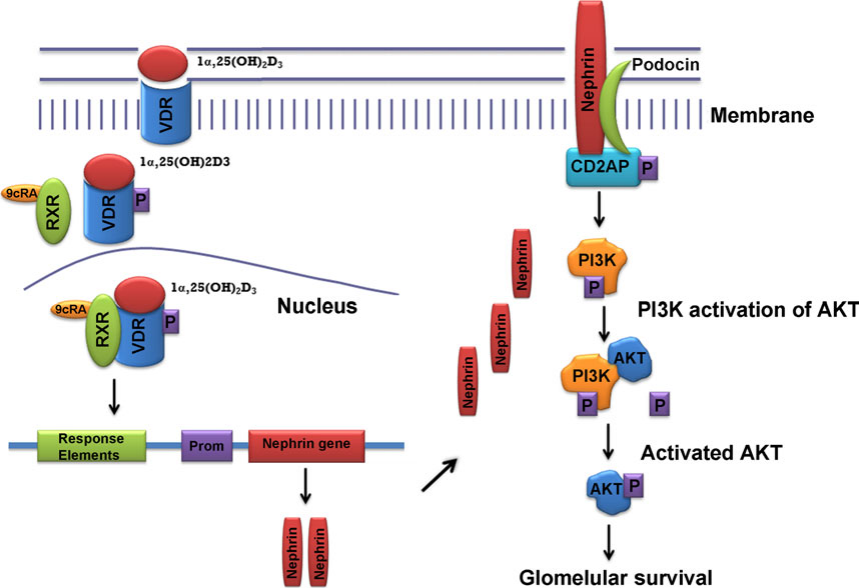
Molecular basis of VDRA beneficiary action through the nephrin signalling pathway. VDRA treatment enhances VDR expression and promotes VDR and RXR co‐localization in the nucleus, leading to increased nephrin expression, which participates in PI3K‐mediated Akt activation thus promoting glomerular survival.

An important finding of the present study was that VDRA treatment rescues podocytes from hyperglycaemia‐induced apoptosis by influencing the nephrin‐PI3K‐Akt signalling pathway (Fig. 8). Calcitriol has also been reported to ameliorate podocyte injury in DN rats *via* the PI3K/p‐Akt signalling pathway 13. In addition, the renoprotective effects of vitamin D3 and its analogues may be inducing alternative pathways leading to the production of monocyte chemoattractant protein 1 (MCP1), as has been suggested for both glomerular epithelial cells 47 and mesangial cells 48.

In summary, the work described herein unravelled a pivotal role for VDRA in glomerular survival signalling, pointing to an anti‐apoptotic function that may contribute to an important inhibitory action on DN progression. Gradual reduction of nephrin expression accompanied by decreased Akt activity due to hyperglycaemia may further affect glomerular survival. Our data suggest an important role for additional factors, calcitriol and paricalcitol which may contribute to attenuation of podocytic damage and/or loss. Further understanding of the mechanisms involved at the molecular level will contribute to the use of these drug/therapeutic targets for the treatment of DN.

## Author contributions

OT performed acquisition of data, analysed and interpreted the data and drafted the manuscript. EFT performed study conception and design, analysed and interpreted the data, performed funding acquisition and critically revised the article. AC Analysed and interpreted the data and critically revised the article. CI analysed and interpreted the data, critically revised the article and performed provision of VDRA. GD performed study conception and design, and acquisition of data, analysed and interpreted the data, performed funding acquisition, drafted the manuscript and critically revised the article.

## Conflict of interest

The authors indicate no potential conflict of interests. This work was partly supported by a grant from Abbvie Pharmaceuticals S.A. (Hellas) that did not have any involvement in designing experiments, collecting, analysing or interpreting data and drafting or submitting the manuscript.

## Supporting information


**Figure S1.** Expression levels of Akt.
