# Supplementary figures and images for: Vitamin D3 ameliorates podocyte injury through the nephrin signalling pathway
Source: J Cell Mol Med. 2017 Jun 29;21(10):2599–609. doi: 10.1111/jcmm.13180 (PMC5618699; doi:10.1111/jcmm.13180)

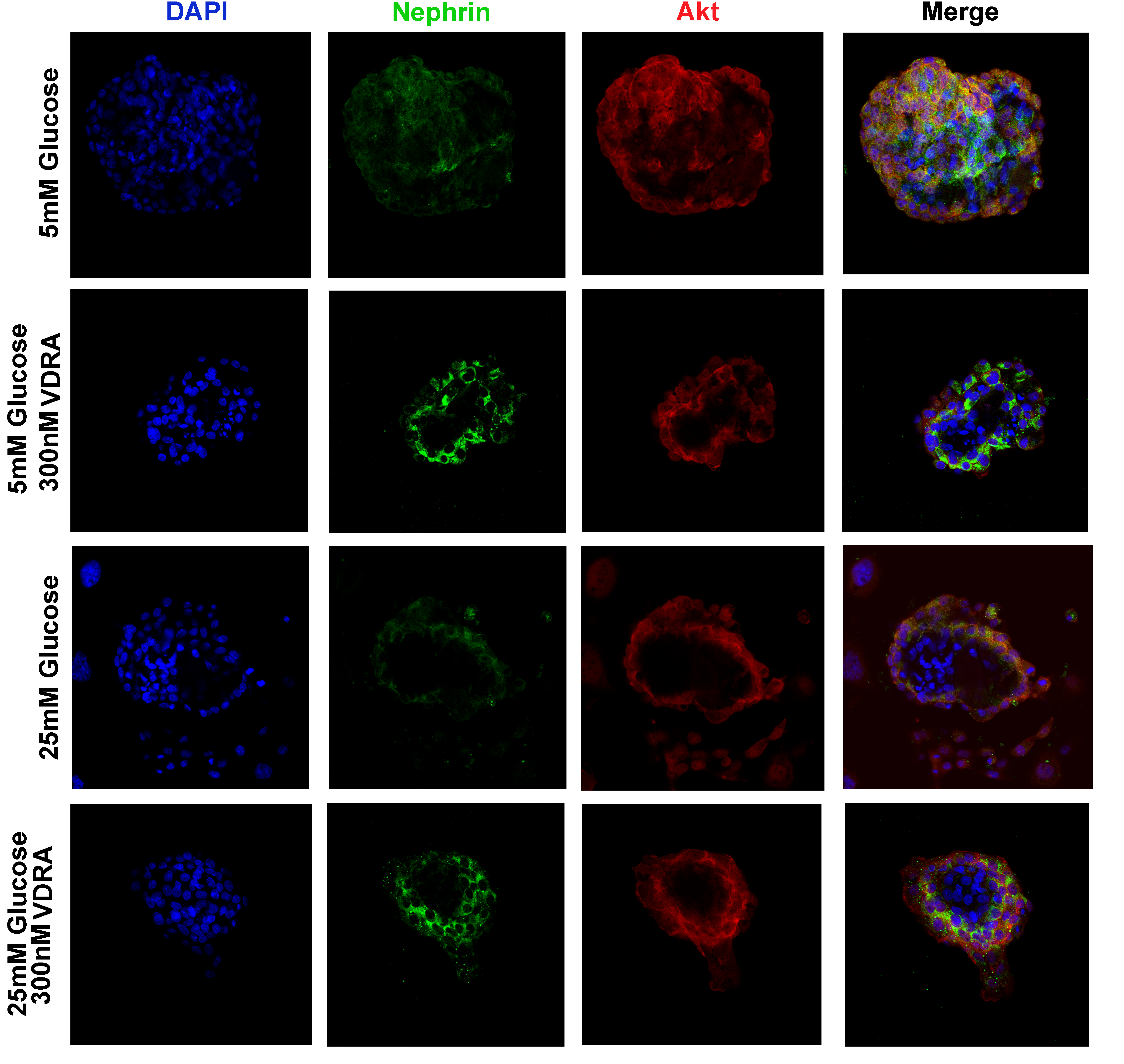

Supplement: Supplementary file 1 — Figure S1. Expression levels of Akt. [file JCMM-21-2599-s001.tif]
